# LowMACA: exploiting protein family analysis for the identification of rare driver mutations in cancer

**DOI:** 10.1186/s12859-016-0935-7

**Published:** 2016-02-09

**Authors:** Giorgio E. M. Melloni, Stefano de Pretis, Laura Riva, Mattia Pelizzola, Arnaud Céol, Jole Costanza, Heiko Müller, Luca Zammataro

**Affiliations:** Center for Genomic Science of IIT@SEMM, Fondazione Istituto Italiano di Tecnologia (IIT), Via Adamello 16, 20139 Milan, Italy

## Abstract

**Background:**

The increasing availability of resequencing data has led to a better understanding of the most important genes in cancer development. Nevertheless, the mutational landscape of many tumor types is heterogeneous and encompasses a long tail of potential driver genes that are systematically excluded by currently available methods due to the low frequency of their mutations. We developed LowMACA (Low frequency Mutations Analysis via Consensus Alignment), a method that combines the mutations of various proteins sharing the same functional domains to identify conserved residues that harbor clustered mutations in multiple sequence alignments. LowMACA is designed to visualize and statistically assess potential driver genes through the identification of their mutational hotspots.

**Results:**

We analyzed the Ras superfamily exploiting the known driver mutations of the trio *K-N-HRAS,* identifying new putative driver mutations and genes belonging to less known members of the Rho, Rab and Rheb subfamilies. Furthermore, we applied the same concept to a list of known and candidate driver genes, and observed that low confidence genes show similar patterns of mutation compared to high confidence genes of the same protein family.

**Conclusions:**

LowMACA is a software for the identification of gain-of-function mutations in putative oncogenic families, increasing the amount of information on functional domains and their possible role in cancer. In this context LowMACA emphasizes the role of genes mutated at low frequency otherwise undetectable by classical single gene analysis.

LowMACA is an R package available at http://www.bioconductor.org/packages/release/bioc/html/LowMACA.html. It is also available as a GUI standalone downloadable at: https://cgsb.genomics.iit.it/wiki/projects/LowMACA

**Electronic supplementary material:**

The online version of this article (doi:10.1186/s12859-016-0935-7) contains supplementary material, which is available to authorized users.

## Background

As previously described, the identification of driver mutations in cancer can be enhanced by considering the position of the mutations on the proteins rather than their simple frequency in cancer cohorts [1]. For this reason, tools that combine frequency of mutations and their position on the genome have been recently developed for the identification of potential drivers in small cohorts of patients to increase statistical power [2–4]. Furthermore, other methods based on network analysis were developed to aggregate mutational information at the level of interaction pathways [5]. Nevertheless, as pointed out in a recent simulation based on saturation analysis on publicly available cancer data, we are still far from a true understanding of the genes mutated in less than 5 % of the patients for almost any tumor type [6]. Due to the lack of the required sample size, methods able to assess the role of rarely mutated genes are needed. LowMACA represents a solution to increase the information content of alteration patterns by summing up mutations on properly aligned amino acids in different proteins belonging to the same family. The accumulation of somatic mutations in specific Pfam domains has been already observed in cancer, introducing the concept of domain landscapes of somatic mutations in addition to the well-known genomic landscape [7–9]. Nevertheless, these approaches only rely on the frequencies of mutated domains in cancer. We enhance this approach by adding the positional information of mutations within the domains, eventually increasing the statistical power of the domain level analysis. With LowMACA, we are able to assess various aspects of somatic mutations at the level of protein families, including clustering in hotspots, conservation of mutated residues, pattern similarity across proteins and co-occurrence or mutual exclusivity among positions resulting significant by LowMACA criteria. In fact, one of the significant improvements over existing methods is the ability of LowMACA to test single driver mutations and not only driver genes. All these unique aspects are illustrated here in the context of the Ras superfamily and in the analysis of a state-of-the-art set of high confidence and putative driver genes [10].

## Implementation

### Software implementation and overview

LowMACA is a computational tool for the analysis and visualization of somatic mutation data in cancer. It allows to properly assess the significance of hotspots of mutations shared across protein families and to show the interconnectivity among mutational patterns via different visualization methods. The software comes as an R package, fully integrated in the R/Bioconductor environment through the use of the AAMultipleAlignment class from the Biostrings library. The multiple alignment is performed with a wrapper around a clustal omega executable [11] or the EBI soap webserver [12]. At the present time, LowMACA is the only tool that allows using clustal omega within R storing results within a Biostrings class. Importantly, the LowMACA package implements a user-friendly GUI built with the shiny package, exploiting the interactive functionalities provided by D3 javascript and google charts plotting libraries. The tool comes with a pre-built annotation package named LowMACAAnnotation, that integrates the information of HGNC [13], UNIPROT [14] and Pfam [15] with the aim of guiding the user through the analysis of highly conserved classes of proteins belonging to common Pfam domains. The LowMACAAnnotation package creates a one-to-one match between UNIPROT canonical proteins and HGNC gene symbols and provides all the Pfam sequences of each protein entry.

LowMACA implements two conceptually different workflows: a Hypothesis Driven workflow and a Data Driven workflow.

The Hypothesis Driven workflow consists of:

1. Selecting proteins belonging to the same family (we suggest Pfam as a guideline).
2. Selecting one or more tumor types and classes of mutations that will be analyzed (see Methods section Input Data).
3. Retrieving mutations from specified cancer samples.
4. Aligning selected sequences along with their mutations
5. Calculating statistics and evaluating significant hotspots with different parameter settings

The Data Driven workflow consists of:

1. Providing a dataset of mutations from a cancer cohort in a format derived from TCGA standard maf files (see Input Data).
2. LowMACA collects all the genes that harbor at least one mutation and aligns their domains according to Pfam. Subsequently, the mutations are mapped on every consensus sequence created (one per Pfam analyzed).
3. LowMACA analyzes the mutational pattern of every protein by itself.
4. The hotspots found at point 2 and 3 are unified in one table and the list of putative driver mutations is presented (detailed information can be found in the package reference manual: http://bioconductor.org/packages/release/bioc/manuals/LowMACA/man/LowMACA.pdf).

### Input data

According to the choice of a Hypothesis Driven or Data Driven workflow, LowMACA requires different kinds of input. In the first case, LowMACA expects as input a Pfam ID of interest (e.g., “PF00001”) and/or gene names, provided as Entrez Gene IDs [16] or HUGO Gene Symbols [13]. In case only a Pfam ID is provided, the LowMACAAnnotation package will look for all the genes that contain the specified domain, otherwise, only the chosen genes are retained. By selecting a Pfam ID of reference, only the portion of the proteins mapping to the Pfam domain will be considered in the analysis. If a set of gene identifiers is selected without specifying any Pfam ID, the entire protein sequences are considered for the analysis. LowMACA admits also the use of non-ambiguous gene aliases. The LowMACAAnnotation package is designed to assign only canonical proteins to the relative gene creating a one-to-one unique match.

LowMACA retrieves mutational data via the R/CRAN package “cgdsr” [17] which queries the Cancer Genomics Data Server (CGDS) hosted by the Computational Biology Center at Memorial-Sloan-Kettering Cancer Center (MSKCC) [17, 18]. Mutation data coming from personal databases can alternatively be used, following the instructions provided within the manual of our R-package. Since LowMACA looks for hotspots of mutations, the package keeps by default only the mutations that modify the protein without altering the reading frame or creating stop codons (collectively identified as missense type mutations) [4]. Other mutation types, such as frame shift InDels, nonsense mutations or splice-site mutations (collectively called truncating mutations), can be retrieved by modifying the parameters. By default, LowMACA will take into account all the tumors present within the cBioPortal [17, 18] repository, but mutations from specific cancer types can be selected.

In case a data driven workflow is chosen, the user has to provide only mutation data. These data are a direct derivative of a common maf file as specified by TCGA and contains the mutations annotated by their gene, their amino acid change, sample of origin and type of mutation. A detailed description can be found in the package reference manual: http://bioconductor.org/packages/release/bioc/manuals/LowMACA/man/LowMACA.pdf.

### Alignment and mapping

Amino-acid sequences selected as described above are aligned using the multiple sequence alignment software Clustal Omega [11, 12, 19]. Although the Pfam database is a comprehensive archive of cross-species alignments, we only refer to human proteins and each clustal omega alignment represents a unique combination of conserved and not conserved residues. Using the original HMM model of the protein family is a limiting factor in this case, as we would lose portions of alignments specific to human proteins only. Moreover, Clustal Omega can handle alignments involving whole protein sequences, rather than only Pfam domains. From the output of the multiple alignment, a consensus sequence including the most represented amino acid found at every position is created that is representative of all the sequences under investigation. The mutations coming from aligned sequences are remapped directly on the consensus with the aim of obtaining a unique mutational profile.

Considering that LowMACA specifically aims at highlighting mutations that fall on conserved residues, two measures of conservation are taken into account at this point. The first one concerns the specific positions of the alignment. LowMACA calculates the Trident conservation score for this purpose [20], which is a mixed measure that encompasses three different aspects of a local alignment:

1. The entropy of the residues at the specific position. The more different amino acids are aligned the less conserved is the position.
2. The chemical similarity according to the substitution matrix BLOSUM62
3. The relative frequency of gaps

The second measure is global and involves the entire sequence. The alignment procedure of the LowMACA engine is delicate due to the fact that including dissimilar sequences in the analysis can invalidate the whole LowMACA workflow. For this reason, sequence similarity for every pair of amino acid sequences is calculated, based on the k-tuple measure [21], and a warning is prompted whenever an amino-acid sequence differs too much from the others (threshold = 0.2).

These measures are a safety net to avoid false positive results due to low quality alignments and become extremely useful if the user decides to perform analysis with sequences not belonging to the same family. LowMACA provides the Pfam based framework as a guideline, but in theory every mutation profile can be compared.

### Statistical model

#### Testing the randomness of the global mutational profile

Once the sequences are aligned and the mutations have been remapped on the consensus sequence, LowMACA measures the information contained in the mutational pattern [4] using Shannon’s definition of entropy

$$ H(X)=-\sum_i^KP\left({x}_i\right)lnP\left({x}_i\right) $$

where $P\left({x}_i\right)=\frac{n_i}{N}$ is the frequency of mutations mapping to the position *i* of the consensus alignment of length *K* and *N* is the total number of mutations.

To statistically assess whether the pattern of mutations significantly differs from randomness, we compare *H*(*X*) with the entropies of a bootstrap of one thousand random profiles. Each random profile is generated according to the following criteria: (i) the random profile has the same length of the consensus sequence generated from the analysis (i.e., *K*); (ii) the number of mutations that map on the random profile is equal to the total number of mutations that map on the consensus sequence (i.e., *N*); (iii) the probability of a mutation to fall onto a specific position of the random profile is proportional to the number of amino acids that map in the corresponding position of the multiple alignment. In this way, the more gaps are found in a position of the alignment, the lower is the probability that a mutation falls in that position in the random model. This last criterion is intended to correct the bias of finding more mutations in more conserved regions of the consensus. We fit the parameters of a Gamma distribution over the empirical distribution of the entropies calculated on the random profiles. This will be considered as the null distribution and used to assign a p-value to the global mutational profile.

#### Testing for the identification of hotspots of mutation

LowMACA is also able to identify significant positions along the consensus sequence, as opposed to the large majority of driver gene identification approaches [10]. The probability that the number of observed mutations *n*_*i*_ on position *i* of the consensus sequence derives from a random pattern of mutations is calculated estimating the per-position null distribution of the number of mutations that are expected to fall on that specific position. The null distribution is modeled using the Gamma distribution whose parameters are estimated from the bootstrapped random profiles generated for testing the randomness of the global mutational profile. A per-position p-value that the observed number of mutations originated from the null distribution is then calculated and p-values of residues that fall onto conserved positions (Trident score > 0.1) are corrected to obtain per-position q-values using the Benjamini-Hochberg procedure for multiple testing correction [22].

### LowMACA output

Using a Hypothesis Driven workflow, LowMACA outputs a detailed report of the mutational landscape of the consensus sequence. It specifies if the entire mutation profile can be considered random (global p-value), and it reports all the mutation hotspots that exceed the random distribution (per-position p-value and relative FDR corrected q-value); see Statistical Model section. Mutations that fall onto significant positions of the consensus sequence can be retrieved in their original position with a reverse mapping provided by LowMACA.

**Fig. 1:**
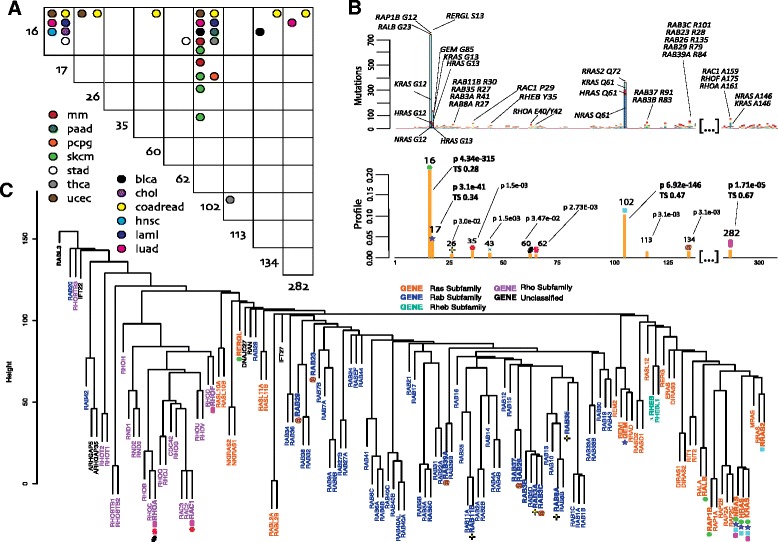
**b** LowMACA results based on the alignment of the Ras superfamily (PF00071). The first barplot reports the most mutated proteins under significant hotspots in their original position. These hotspots are also highlighted in the second barplot with colored symbols. Labels in the second barplot report the position of the consensus, the FDR corrected p-value and the trident score of conservation (TS). The TS is reported only for hotspots identified in the alignment of all the 133 family members. Both barplots are truncated on non-informative positions. **a** The panel shows a plot representing the mutual exclusivity between mutations that fall in the same position of the global consensus alignment. Significant patterns are highlighted with the color corresponding to the tumor type where the mutual exclusivity was found. We consider mutually exclusive the pairs with a corrected p-value below 0.05 using the R package cooccur. **c** The dendrogram is built on hamming distances between all human sequences of the Ras superfamily aligned via clustal omega. Genes that belong to the same subfamily, as described in [Hall, 1998], are represented with the same color. Significant hotspots (under gene names) are represented with the symbols used in **b**

The mutational profile can be visualized with many LowMACA methods. These plotting capabilities are considerably extended through the GUI. The interactivity that this implementation allows is particularly useful to observe the dynamic connections among mutational profiles of different proteins. The following plot types are offered by the package:

1. A stacked barplot that specifies the relative frequency of mutation per sequence in each position (in the GUI this plot has interactive features). This representation also includes a graphical view of the trident score and a logo plot of the most represented amino acids at every position.
2. A Protter style plot [23] that represents the possible secondary structure of the consensus sequence with the significant positions found by LowMACA highlighted in red.
3. An interactive network plot in which the nodes represent the single sequences and the edges are drawn based on the number of shared mutated residues. The thicker are the edges, the more positions are in common. This representation provides an overview of the similarity among sequences in terms of mutational profile.
4. A heatmap of mutual exclusivity and co-occurrence of mutations at the entire sequence level and at single position level implemented with the R package co-occur [24]. For example, it can represent mutual exclusivity between mutations in *KRAS* and *NRAS* and between *KRAS* G12 and *NRAS* G12 positions (see Fig. 1a).

The last two functionalities are only available through the LowMACA GUI.

In a Data Driven workflow, the output is represented in a very similar way, but LowMACA takes care of analyzing all the Pfam domains through the mutations in the genes provided by the user in a single procedure. Every Pfam analysis can become a new LowMACA object and it can be viewed from a descriptive point of view as shown above in the Hypothesis Driven workflow.

## Results

Our results are reported in three different sections. The first analysis is aimed at demonstrating the core concept of LowMACA using a known oncogenic family. Starting from the cancer genes *KRAS*, *NRAS* and *HRAS (*that we will name *RAS trio)*, similar in structure and mutational profile, we seek to extend this conservation to all the Ras superfamily members (in total, 133 different proteins belonging to the PF00071). We demonstrate how LowMACA can be used to show the oncogenic potential of different positions of the family and to encompass new putative driver genes through the sharing of conserved mutations. We also evaluated mutual exclusivity of mutations that fall in specific positions of the consensus alignment (Fig. 1a and Additional file 1: Figure S2). Moreover, by collecting all the observed mutations that fall in PF00071, we show that LowMACA hotspots fall in positions that are expected to be damaging by 8 different predictors of phenotypic effect. Although LowMACA predictions and mutation damage assessments are in agreement with the other predictors, our tool is more specific in assessing driver mutations against a gold standard of known cancer driver mutations and disease associated mutations (see Additional file 1: Figure S1).

The second analysis is aimed at assessing the state-of-the-art in driver genes at a domain level. By taking a curated list of high confidence drivers (HCDs) and a list of candidate driver genes (CDGs) derived from 5 different bioinformatic tools [10], we study the relationships in terms of common mutations among these genes. We show that 40 % of all the HCDs share at least one domain with a CDG defining and expanding the same concept illustrated in the Ras example. Mutations that fall in known driver genes are shared both by other known drivers (like the tyrosine kinases *EGFR*, *BRAF*, *FLT3* and *JAK* family) but also by less frequently mutated genes with a similar structure (like the receptor L domain genes *ERBB2* with *ERBB4*).

The third analysis shows, as a negative control, that silent mutations do not have the propensity to show significant pattern of mutations.

### Ras superfamily analysis

We aligned and summarized the mutational landscape of the Ras superfamily, defined by PF00071. This Pfam represents a large family of small GTPases that can be grouped in different subfamilies with specific biological characteristics [25].

We performed our analysis in two steps. First, we aligned all the mutations of the entire family encompassing 133 sequences. Second, we performed the same analysis dividing the mutations by the four main subfamilies: 1) Ras subfamily, involved in cell proliferation [26], 2) Rheb subfamily, involved in neural plasticity [27], 3) Rho subfamily, involved in cytoskeletal morphology [28] and 4) Rab family, involved in cell trafficking [29].

Analysis of the entire family found significant hotspots in the consensus alignment in positions 16, 17, 102, and 282, as highlighted in Fig. 1a. In this analysis, we discuss genes that have at least two mutations in any of the identified hotspots. These mutations are well conserved in the superfamily but appear mainly represented in the Ras subfamily. The main representative members of this proto-oncogenic subfamily are the known cancer genes that compose the *RAS trio.* Their mutations G12, G13, Q61 and A146, considered important drivers in many cancers [30], map on the hotspots identified above. These three proteins share over 90 % of sequence identity in the domain and are the most represented in terms of absolute number of mutations in these positions.

Hotspots found in position 16 of the global alignment harbor mutations on residues G12 of *RAP1B,* on residue S13 of *RERGL* and G23 of *RALB*, which align with G12 of the *RAS trio*, while position 17 aligns with mutations on G85 in *GEM,* which aligns with G13 of the *trio.* Even if these proteins, (excluding the *trio*) are very rarely mutated, LowMACA identifies their alterations as putatively oncogenic (Fig. 1b). All these proteins belong to the Ras subfamily, but a particular exception is represented by *RERGL* that harbors a recurrent S13F mutation: this protein is considered part of the Ras subfamily but its sequence is very distant from the *RAS trio* (Fig. 1c) and for this reason should be analyzed separately.

Another highly conserved mutation is located in the aligned position 102 that corresponds to mutations in Q61 in *NRAS* prevalently and is one of the residues involved in the binding function of all the Ras family members to GTP [31]. LowMACA analysis highlighted mutations aligned in position 102 also in other Ras members, in particular Q72 mutations in *RRAS2*. This gene has been extensively analyzed at the transcriptional level but remains poorly investigated regarding the mutational context [32]. *RRAS2* has a role in pathways activated by the *RAS trio*, however, while the *trio* exerts its pro-proliferative activity via the activation of the Raf-ERK pathway of MAP kinases, *RRAS2* activates this pathway poorly as it does not recruit Raf1 [32]. Following the observation of several Q72 mutations in RRAS2, one might speculate on a possible activation of this gene in the same way as Q61 activates *NRAS*.

Position 282, corresponding to an alanine in 146 in the RAS trio, represents a completely different case. This hotspot is extremely well conserved in all the members of the superfamily and represents the only case of a significantly mutated residue shared by two different Ras subfamilies (Ras and Rho). This mutation does not impair the affinity with GTP (like G12/13 and Q61) but rather seems to have an effect on the GTP-Ras steady-state levels as reported by experimental assays [30]. *RAC1*, *RHOA* and *RHOF* emerge as putative oncogenes by this analysis, sharing mutations in this position. Among these, *RAC1* and *RHOA* are already present in the Cancer Gene Census [33], adding confidence to the hypothesis that also *RHOF* might play a role in cancer. Moreover, relatively elevated levels of RHOF were observed in lymphomas derived from the germinal centre [34].

Hotspots identified in the previous analysis correlate well with sequence similarity based on hamming distance (Fig. 1c). For example, the aforementioned hotspots 16, 17 and 102 belong specifically to the Ras subfamily, identified in orange in the dendrogram. This subfamily harbors two glycines in position 16 and 17 that are not shared by the entire superfamily. In fact, the 16/17 glycines can be substituted by the couple serine/glycine (Rab subfamily) or the couple glycine/alanine (Rho subfamily) [25]. The Rheb subfamily instead, composed of just two genes *RHEB* and *RHEBL1*, does not conserve any of the two marker residues and carries a distinctive leucine in position 16. By analyzing mutations that fall individually in each of the four subfamilies, we were able to identify new putative oncogenes and new hotspots of mutation. In order to keep the reference with positions identified with the global analysis, we maintained the full alignment of all the proteins of PF00071 and then subset the genes of interest according to the four subfamilies (this alignment parameter is called “datum” in the LowMACA package).

The analysis of the Rab subfamily (mostly represented in the central portion of the dendrogram in Fig. 1c) highlights three new hotspots and 11 new putative oncogenes. Among these, *RAB29* harbors 4 mutations in position 134 of the alignment that are predicted to be damaging by most of the functional predictor tools used in Additional file 2: Table S5 (R79W in Colorectal cancer and R79L in Lung adenocarcinoma). The involvement of members of this subfamily in cancer has been widely demonstrated [35].

The analysis of the Rho subfamily allowed the identification of new hotspots, which are mainly represented by *RAC1* and *RHOA. RAC1* marks a single hotspot found in position 35 corresponding to mutations of the residue proline 29 (*RAC1* P29). According to the most recent literature, P29 results altered in approximately 3.9 % of TCGA skin cutaneous melanoma patients [36] suggesting that *RAC1* is a melanoma oncogene. The biological significance of the *RAC1* P29 mutation remains unclear, although authors demonstrated that the mutation could destabilize the *RAC1* inactive GDP-bound state in favor of its active GTP-bound state, creating a gain-of-function oncogenic event [36]. In fact, the expression of *RAC1* P29S in sensitive *BRAF*-mutant melanoma cell lines confers resistance to treatment with *RAF* inhibitors [37]. Moreover, the P29S mutation has been reported in several cancers such as head and neck tumors [38] and breast tumors [39]. The hotspot 35 is also shared by other Rho subfamily members: *RAC2*, *RHOT1*, *RHOC*. Even though one single mutation was found for each gene in our dataset, this position is extremely well conserved (a proline is present in all four genes) and all the mutations were found in melanoma patients without a *RAC1* P29 mutation (Additional file 2: Table S5).

The mutational hotspots 60 and 62, respectively corresponding to glutamate 40 and tyrosine 42 in *RHOA*, were observed in seven tumors (six head and neck, one breast) and affect the effector domain of *RHOA* [6]. *RHOA*, is considered a gene encoding a protein that is clearly involved in cell proliferation [6]. As for the case of *RAC1,* also *RHOA* shares its hotspots with other Rho subfamily members (these results are not reported in Fig. 1 since only one mutation was found in our dataset). These genes include *RHOH* E39K for hotspot 60 and *RHOC* Y42C and *RAC1* Y40S for hotspot 62. Both positions are still well conserved in the subfamily (Additional file 2: Table S5).

The analysis of the Rheb subfamily shows a significant number of mutations that fall in the hotspot 43. These mutations are mostly represented by Y35N hosted by *RHEB* and found present in Kidney Renal Clear Cell and Uterine Corpus Endometrioid Carcinomas in TCGA patients. Moreover, authors observed that mutations of *RHEB* (Y35N/C/H) increase phosphorylation of endogenous substrate S6 kinase (S6K1) of the mTOR signaling pathway [40], a protein kinase that plays key roles in cellular regulation [41]. For the presence of the Y35N mutation, *RHEB* was recently highlighted as a novel cancer gene involved in cell proliferation [6], and cancer associated mutations in *RHEB* inducing mTORC1 activity have been reported [40]. The only other member of the subfamily (*RHEBL1)* shares a Y35H mutation in the same hotspot in one melanoma case in our dataset.

#### Mutual exclusivity analysis

In order to corroborate LowMACA results reported above, we performed mutual exclusivity analysis on significant mutations and hotspots. Mutual exclusivity between mutations on genes of the same pathway is a critical measure to assess if the pathway is relevant for cancer. The reason is that after the first mutation occurs, there is no selective pressure for a second mutation in another gene of the same pathway [42]. While generally performed gene-wise [43], the particular characteristics of LowMACA allow us to extend this concept to mutations that map on conserved residues within Pfam domains. If a putative driver mutation is found to be mutually exclusive with a known driver, its significance is enhanced as it possibly exerts the same function in cancer. We implemented mutual exclusivity analysis using the R package *cooccur* for a genomic analysis [24] stratifying mutation data by tumor type.

Our results revealed that hotspots in positions 16, 17, and 102 cover the large majority of mutually exclusive patterns (Fig. 1a). This is a confirmation of the known exclusivity pattern of the mutations in *KRAS* and *NRAS* even among different positions within the genes themselves (Additional file 1: Figure S2, right panel). In general, mutations in position 16 and 102 can be seen as a signature of two types of cancer: colorectal, characterized by *KRAS* G12, and melanoma, characterized by *NRAS* Q61 (Additional file 1: Figure S2, left panel) [30]. These two highly frequent mutations allowed us to infer a possible driver role for less frequent mutations. For example, mutations in positions 26, 60 and 134 in colorectal cancer are mutually exclusive with position 16. Both hotspots are supported by this analysis in the Rab and Rho subfamilies. Similarly, position 102 is mutually exclusive with 26 and 35 in melanoma and 113 in thyroid cancer, further supporting the role of the aforementioned subfamilies.

### Analysis of driver genes: comparison with available tools

In this section, we analyzed the state-of-the-art driver genes identified with different bioinformatics tools under the lens of the protein families they belong to. In particular, we focused our attention on the 435 genes identified by a unifying approach as presented in [10]. In this study, driver genes are divided in two categories, 291 High Confidence Driver (HCDs) and 144 Candidate Driver Genes (CDGs), according to several criteria, which include: 1) the number of bioinformatic tools that identify the gene as potential driver (5 tools were taken into consideration), 2) if the gene belongs to a list of manually curated cancer genes as provided by the Cancer Gene Census (CGC) [33], 3) if the gene belongs to the same pathway in the KEGG database [44]. With this analysis we want to add address two questions: what Pfam domains are contained in driver genes and what are the candidate driver mutations shared between HCDs and CDGs according to LowMACA criteria.

**Fig. 2:**
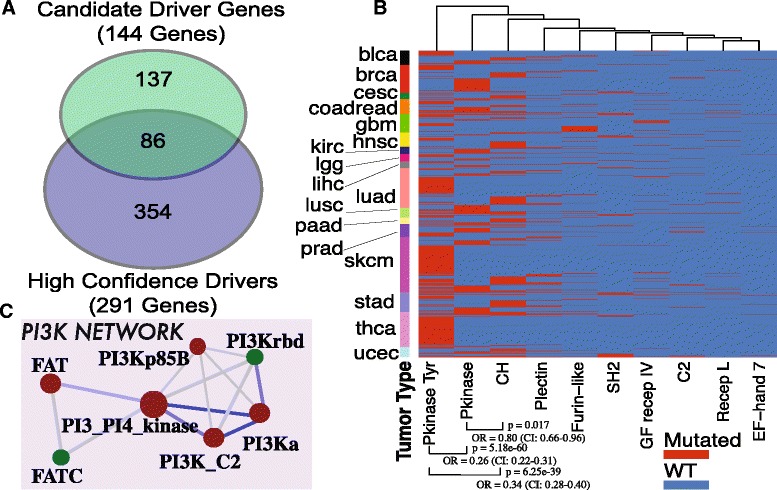
**a** Venn diagram of the represented Pfam domains in the list of 291 high confidence drivers and 144 candidate drivers. A total of 577 different Pfam domains are covered by these genes with 86 Pfam domains shared between the two lists. **b** Heatmap representation of significant Pfam domains in the “Kinase” network. Every row represents a patient of 17 different tumor types. A strong mutual exclusivity between tyrosine kinases, kinases and CH domain is shown. **c** PI3K networks in driver genes. Every circle represents a distinct Pfam domain and the size represents the number of genes that contain the specified Pfam domain. Color indicates if significant hotspots were found in the LowMACA analysis (red is significant, green is not significant). Two domains are connected if they are found together on the same gene/protein. Edge thickness represents the number of genes that harbor both Pfam domains at the vertices (minimum 2). Blue color indicates mutual exclusivity and orange depicts significant co-occurrence

Since we are considering missense mutations, most of the tumor suppressors contained in the driver gene list will not be covered by LowMACA. In fact, tumor suppressors tend to lose their function during tumorigenesis and mutational landscapes are typically represented by sparse truncating mutations all over the gene body [1]. In this case, no clear clusters can be seen at single amino acid level because for a gene to lose its protein function there are generally no preferential positions. Furthermore, many tumor suppressors are singletons in the Pfam database, in the sense that their main domain can only be found in the genes themselves or in few other members (e.g., P53 Pfam, PF00870, is only shared by three genes *TP53*, *TP63*, *TP73*, Suppressor *APC*, PF11414, belongs to *APC* and *APC2* only). Nevertheless, highly mutated tumor suppressors like *TP53*, *VHL*, *RB1 ARID1*, *PTEN* and *APC* form actual hotspots that resulted significant in the LowMACA analysis (Additional file 3: Table S2–3, reference list of tumor suppressors derived from [45]). Other known tumor suppressors such as *WT1*, *CEBPA* or *CDKN1A* are instead missed by our analysis. The case of *TP53* is particularly interesting as it tends to form clusters of missense mutations specifically on its *P53* domain that probably exert oncogenic or dominant negative functions [4]. The fact that some tumor suppressors are identified and some are not depends in large part from the frequency of mutations. As the frequency increases, the sensitivity is enhanced and preferential positions of distruption emerges. Preferential mutation spots, even in tumor suppressors, are generally explained by possible dominant negative or oncogenic signature of certain tumor suppressors [46, 47] but also by a higher susceptibility to carcinogens of certain codons in these genes compared to other codons [48] 577 different Pfam domains are covered by the driver gene list, approximately one tenth of the entire Pfam-A database: 440 in the HCD list, 223 in the CDG list and 86 in common (Fig. 2a, Additional file 3: Table S1). To assess whether the overlap between the Pfam domains contained in the lists of CDG and HCD is greater than expected, we randomly sampled the same amount of genes that are contained in the two lists and measured the overlap of the contained Pfam domains. On average, we found a smaller overlap (57 ± 7), but also a smaller number of Pfam domains in the CDG-sized samples (194 ± 11) and in the HCD-sized samples (355 ± 15). We conclude that driver genes contain more domains than the rest of the other human genes (*p* = 7e–9 and *p* = 4e–3 for HCD and CDG, respectively, via z-test) but their overlap is not significant (*p* = 0.38 via chi-squared test). The first two significant p-values can be interpreted as an expected enrichment in functional portions for the driver gene list compared to the rest of human genes. The not significant overlap instead could be interpreted as an enrichment of singletons caused by the great amount of tumor suppressors but also as a lack of connections between the two lists from the domain point of view.

We performed LowMACA analysis in order to find significant hotspots of mutations at two different levels: 1) all the domains were analyzed by aligning the specific sequences of each HCD and CDG that harbors them and 2) the entire protein was scanned for hotspots considering just its sequence, without any alignment. The second analysis was performed to look for protein-specific hotspots that could be found outside of the Pfam domains and to prevent the exclusion of genes that are not considered by the Pfam-A database (e.g., *WT1*). Obviously, conservation plays no role in this case.

Our results identified hotspots of mutation in 11 out of the 137 Pfam domains that were found only in CDG (8 %), 32 out of the 86 Pfam domains that were shared both by CDG and HCD (37 %) and 188 Pfam domains that were found only in HCD (53 %) (Additional file 3: Table S3). The higher number of domains that were found significant in HCD compared to CDG reflects the increased number of mutations in each category. Overall, 52 out of 144 candidates (36 %) and 177 out of 291 drivers (60 %) are supported by LowMACA analysis, either by single sequence analysis or Pfam analysis (Additional file 3: Table S2). Hotspots that are supported with single-sequence analysis (found in 140 genes for HCDs and in 35 genes for CDGs) highlight genes that do not need further support from Pfam companion genes for their identification. Pfam analysis added support to further 37 driver genes and 17 candidates. Compared to the number of genes identified on single sequences, the analysis of the Pfam domains increased the number of identified genes by 26 % in HCD and by 50 % in the CDG categories, reflecting the fact that LowMACA is particularly useful in identifying genes that mutate at low frequency. In fact, the major gain is found in the CDG category whose genes are typically less frequently mutated.

To better characterize recurrence of Pfam domains within the CDG and HCD genes, we built a group of networks where vertices are Pfam domains and edges connect domains that are included together in at least two protein sequences (Additional file 1: Figure S3). The three main connected graphs are represented by the “PkinaseTyr” network, the “PI3K” network (Fig. 2c) and the “HelicaseC” network, which were named after their main hub.

The “PkinaseTyr” network encompasses major oncogenes like *BRAF*, *EGFR*, *FLT3* and *ERBB2* for PF07714 (Pkinase_tyr, Additional file 3: Table S3 highlighted in green) and *STK11*, *CHEK2,* MAPKinases (*MAP3K1*/*3*/*4*) and activin receptors (*ACVR1B*) for PF00069 (PKinase, Additional file 3: Table S3 highlighted in yellow). We specifically analyzed the 10 domains which resulted significant with LowMACA and represent them as a heatmap (Fig. 2b): mutated subjects in at least one of the Pfam sequences are depicted in red, while subjects with a wild type domain are depicted in blue. For many tumor types, in particular bladder (BLCA, in black), breast (BRCA, in red) and colorectal (COADREAD, in orange), a clear mutually exclusive pattern is visible, where subjects with mutations in Pkinase have a wild type tyrosin kinase and vice versa (*p* = 5.18e–60, Odds Ratio 0.26 under Fisher exact test). In glioblastoma (GBM, in green), the majority of patients have a mutation on the Furin-like domain (PF00757, Additional file 3: Table S3 highlighted in light blue), mutually exclusive with tyrosine kinases. The most studied missense mutation in this tumor type is in fact *EGFR* A289V/D/T, known for being resistant to anti-EGFR inhibitor used in lung cancer [49]. This alanine residue is perfectly conserved within the Furin-like domain among other epidermal growth factor genes and appears mutated also in *ERBB2* and *ERBB4*, although not in glioblastoma.

The “HelicaseC” network encompasses genes of various families, which are not strictly connected to each other at the functional level. The Helicase_C domain (PF00271, Additional file 3: Table S3 highlighted in red) is the largest significant member of this module and encompasses HCDs as *CHD4*, *SMARCA4* and *ATRX* with two highly conserved arginine residues mutated at low frequency in various tumor types. These mutations affect the corresponding arginine of *CDH7*, *SMARCAD1* and *DDX3X*, which are considered as candidate drivers by the analysis of Tamborero and colleagues [10].

The “PI3K” network is instead a strictly interconnected module with a strong degree of mutual exclusivity between the domains that compose it (blue edges in Fig. 2c). The mutations in these Pfam domains belong for the large majority to three main HCDs (*PIK3CA*, *PIK3CB* and *PIK3CG*). In particular, *PIK3CA* is one of the most mutated genes in many types of cancers. The most relevant mutations appear to be in position 24, 27, and 28 of the multiple alignment of PF00613 (PI3Ka domain) that correspond to E542, E545 and Q546 in *PIK3CA* (Additional file 3: Table S4 highlighted in purple). These mutations can be found conserved also in the other two HCDs at low frequency and a similar role has been already assessed for *PIK3CB* [50]. As we have shown, the overlap between Pfam domains in HCDs and CDGs is not significantly higher than expected from random sampling. This suggests that the current concept of driver genes could be biased due to inappropriate consideration of infrequently mutated genes within the same family. For this reason, we decided to extend our analysis to other possible candidates not present in the list of Tamborero *et al.* [10] in the same way as we did for the Ras family. We thus analyzed all the proteins within the following Pfam domains: PF00794 (PI3K_rbd) PF00792 (PI3K_C2) PF00454 (PI3_PI4_kinase), PF02192 (PI3K_p85B) and PF00613 (PI3Ka). These domains are all shared by the 3 aforementioned HCDs and encompass the majority of their mutations. We found low frequency mutations in *PIK3C2A*, *PIK3C2G* and *PIK3CD,* other members of this kinase family, which were never considered as potential driver candidates before (Additional file 3: Table S4, ranked as New Candidate Driver Gene, NCDG). The first two genes belong to the class II of PI3Ks and their role in human diseases is still unclear [51]. *PIK3CD*, instead, belongs to the same class I of *PIK3CA/B/G* and has been found amplified or overexpressed in cancer [52].

### Analysis of silent mutations

We run as a negative control a LowMACA analysis using a database of silent mutations on the Pfam domains which were involved with a major role in the previous sections: Ras supefamily (PF00071), Pkinase_tyr (PF07714), Helicase_C (PF00271) and PI3Ka (PF00613). This analysis is aimed at assessing whether non-random pattern emerge from silent mutations. We downloaded TCGA data from TCGA original repositories and performed the analysis on this subset since the cBioportal database exclude silent mutations. The analysis of 676, 1144, 216 and 37 silent mutations that fall on the Ras, Pkinase_tyr, Helicase_C and PI3Ka, respectively, do not show any significant hotspot. On the contrary, 5 hotspots are identified in Ras domain, 10 in Pkinase_tyr, 2 in Helicase_C and 3 in PI3Ka when analyzed with non-silent mutations (canonical analysis) (Additional file 1: Figure S4).

## Discussion

We developed LowMACA, a software aimed at characterizing low frequency mutations involving specific residues within the consensus sequence of protein families. LowMACA maps the mutations observed in different members of a protein family to the multiple alignment of the family members. The resulting consensus protein is suitable to summarize the mutation patterns of different proteins and increases the amount of information on functional domains and their possible role in cancer. All the mutations selected by LowMACA frequently fall upon specific positions of the consensus protein and these can be considered as “highly conserved” in cancer.

Moreover, we have identified patterns of statistically significant mutual exclusivity (mutex) among the identified mutations. The presence of these patterns helps to clarify the meaning of all the mutations belonging to specific pathways indicating exclusive roles of the involved genes in cancer. For example, the mutex analysis between *RAC1* and *NRAS* in skin melanomas (Additional file 1: Figure S2) confirms the relevance of the role of *RAC1,* which is co-mutated with *NRAS*, in gain-of-function oncogenic GTP mediated events. The *RAC1* P29L mutation has been experimentally expressed in C. Elegans neurons displaying defects in axon guidance and branching errors that were not seen in equivalent transgenic lines expressing wild-type *Rac1*. Loss of function of the *Rac1* gene did not show any pattern of alteration of axon guidance, demonstrating that *Rac1* P29L is a gain of function mutation [53]. These results suggest that a sort of “code switch” between mutations in *NRAS* and in *RAC1* occurs, probably generating different patterns of cell migration. Translating the experimental observations concerning *RAC1* from a neuronal system to cancer is not straightforward. However, it is tempting to speculate that cancer can orchestrate a complex mechanism of choices depending on the environmental context where it develops. The mutex analysis between Rho members and the *RAS trio* in cancer represents an example of how one out of the many mechanisms underlying cell growth and metastatic processes can provide a selective advantage to cancer cells.

The identification of mutex patterns concerning other proteins belonging to the Ras family suggests that beyond *KRAS*, *HRAS* and *NRAS* other minor genes, such as *RRAS2*, could play a “Ras-like” role in promoting pro-proliferative activity via the activation of the Raf-ERK pathway of MAP kinases [32] in uterine and cervical cancers (Additional file 1: Figure S2). This finding supports the hypothesis that *RRAS2* has a vicariant role in wild type *KRAS* cancers. Other mutual exclusivities have been observed between *HRAS* and *RHOA*, in head and neck squamous cell carcinoma (HNSC) and between *DIRAS2* and *KRAS* in colorectal cancer. The phenomenon by which minor proteins in a family domain can harbor the “same” mutations harbored by known drivers is observable also in other Pfam domains encompassed in the PI3K family. These findings highlight a possible role of minor members of this kinase family in cancer (e.g., *PIK3C2A*, *PIK3C2G* and *PIK3CD)*. LowMACA allows focusing on this phenomenon and helps formulating a possible explanation: cancers cells that gain a selective advantage from major driver mutations in one type of cancer may gain a similar selective advantage from corresponding mutations in closely related proteins in other types of cancer where the related protein plays a prominent role due to tissue specific differences in gene expression or environmental constraints such as exposure to therapeutic agents. In extending LowMACA analyses to other Pfam domains we also demonstrated the existence of liaisons among genes considered high confidence drivers with other genes that are considered candidate drivers. The presence of low-frequency mutations in *ERBB2* and *ERBB4* that correspond to known driver mutations in tyrosine kinases such as *EGFR*, *BRAF*, *FLT3* and *JAK* further strengthens this concept.

Nevertheless, Ras subfamilies also show specific hotspots that reflect the subtle differences played by genes of each subfamily in cellular homeostasis. The Rho subfamily genes have roles in regulating cytoskeletal dynamics and deregulation of Rho proteins contributes to tumorigenesis and metastasis, while Ras subfamily proteins mainly function in regulating cell proliferation [25].

LowMACA is intended as an algorithm that emphasizes low-frequency mutations in genes containing a Pfam domain. Nevertheless, we cannot generalize this concept to all driver genes. For example, genes such as *TP53*, *VHL*, *RB1* or *APC*, show distinct patterns of somatic driver mutations that are not shared by other members of their family (like *TP63* and *TP73* or *APC2*). These tumor suppressors should be considered as singletons and this characteristic underlines the difference between tumor suppressors and oncogenes. Thus, LowMACA is particularly useful for the identification of gain-of-function mutations in putative oncogenic families.

## Conclusion

LowMACA emphasizes the role of genes mutated at minor frequency in cancer, which are often neglected by current analyses. The possibility to classify patients associated to signatures of low-frequency mutations identified by our software represents a promising route for future work. At the same time, a more accurate classification of driver genes may shed light on molecular mechanisms underlying cancer that until now were not yet considered.

## Availability and requirements

**Project name**: LowMACA.

**Project home page**: http://www.bioconductor.org/packages/release/bioc/html/LowMACA.html

LowMACA is also available as a GUI standalone downloadable at: https://cgsb.genomics.iit.it/wiki/projects/LowMACA

**Other requirements**: R 2.10 or higher; LowMACA comes with an accompanying annotation package (LowMACAAnnotation), downloadable from: http://www.bioconductor.org/packages/release/bioc/html/LowMACAAnnotation.html.

**Operating system(s):** Platform independent.

**Programming language:** R

**License**: GPL-3

## Additional files

Additional file 1:
**It contains detailed results of our analyses in Figures S1-S4.** (PDF 1180 kb) Additional file 2:
**It contains detailed results of our analyses in Table S5 and S6.** (XLSX 232 kb) Additional file 3:
**It contains detailed results of our analyses in Table S1-S4.** (XLS 265 kb)

## Abbreviations

CDG: candidate driver genes
CGC: cancer gene census
GUI: graphic user interface
HCD: high confidence driver genes
LowMACA: low frequency mutations analysis via consensus alignment
NCDG: new candidate driver gene
Ras trio: the cancer genes *KRAS*, *NRAS* and *HRAS*
TCGA: the cancer genome project
