# Supplementary material for: LowMACA: exploiting protein family analysis for the identification of rare driver mutations in cancer
Source: BMC Bioinformatics. 2016 Feb 9;17:80. doi: 10.1186/s12859-016-0935-7 (PMC4748640; doi:10.1186/s12859-016-0935-7)
Supplement: Additional file 1: — It contains detailed results of our analyses in Figures S1-S4. (PDF 1180 kb) [file 12859_2016_935_MOESM1_ESM.pdf]

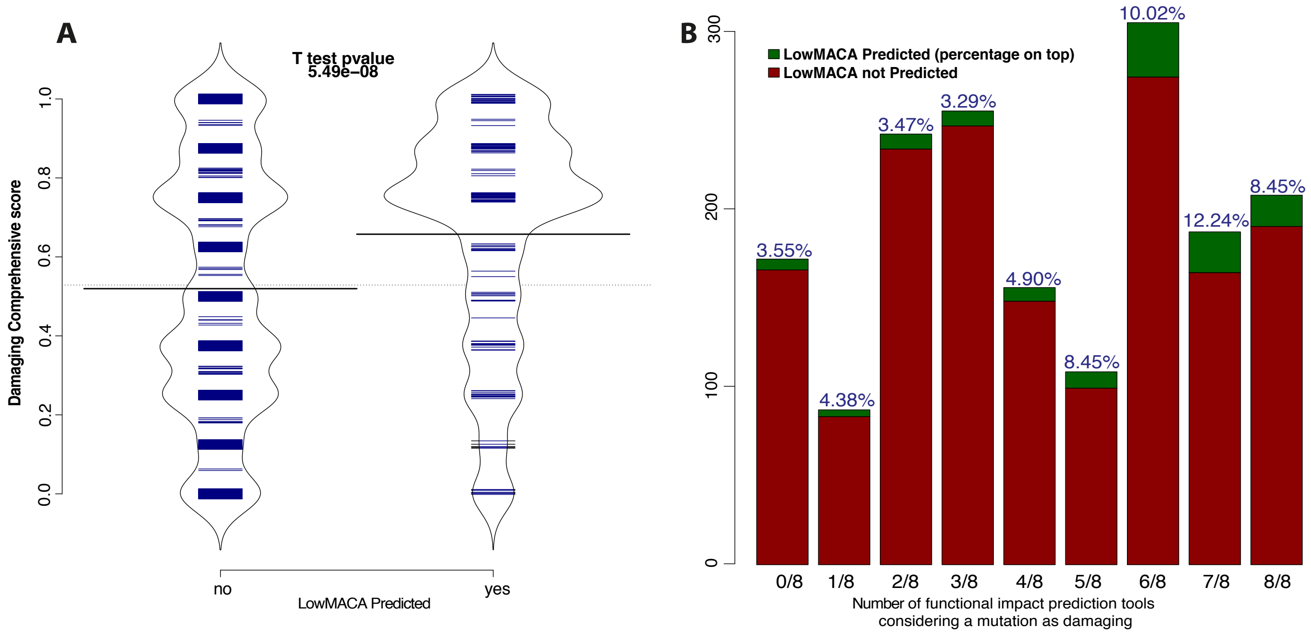

Figure S1

LowMACA results on RAS superfamily (PF00071) compared with 8 functional impact predictive tools summarized in the dbNSFP (Data Base of human Non-Synonymous SNVs and their Functional Predictions)[1]. We retrieved every amino acid substitution occurring in the RAS superfamily from the cBioportal database (more than 10'000 different samples)[2, 3] and we annotated our predictions (if a mutation falls under a significant hotspot of LowMACA, as presented in the Ras superfamily analysis in section Results). The 2294 unique substitutions found in PF00071 cover 2264 positions and 130 proteins of the 133 RAS superfamily genes (Table S5). LowMACA predicts as significant 150 mutated residues under 11 hotspots (Figure 1B), which correspond to 215 different substitutions. We also annotated this dataset including the predictions of functional impact from 8 different tools using ANNOVAR [4]. These tools include PolyPhen2 [5], Mutation Assessor [6], Mutation Taster [7], SIFT [8], MetaLR [9], LRT [10], FATHMM [11] and RadialSVM (http://genomics.usc.edu/software/11-icages) and are aggregated in the dbNSFP database. The functional impact of an amino acid variation can be assessed in many different ways (across species conservation, stoichiometric similarity between original and substitute amino acid, change of protein conformation etc.), but all the algorithms share a similar output, assessing if a mutation could be considered "tolerated" or "damaging". We summarize this information as a damaging comprehensive score: the proportion of tools that predict the variation as damaging, ranging from 0 (all prediction as "tolerated") to 1 (8 out 8 prediction of damaging mutation). In panel A, we show how the 2264 positions are classified in terms of this damaging score. This score is calculated on the actual amino acid substitution and not on the position, so in case there are more possible variations, the median damaging score is considered (like in the case of *KRAS* G12 that can be substitute by V, A, K and other amino acids and a unique damaging score exists for every change). There is a significant difference (p-value of the two tails t-test = 5.49e-08) between the score calculated on the positions not considered by LowMACA and those that fall under LowMACA hotspots. This can be interpreted as a positive concordance between the LowMACA predictions (based on actual data) on the RAS family and their impact on the protein they belong to as calculated by the dbNSFP tools. This simply means that in cancer, the most frequent mutations are also the most damaging. In Panel B, we show how the predictions of LowMACA are distributed. The majority of our predictions fall beyond the majority voting of the tools (5/8 and higher). The same plot in absolute values is presented in Table S6. To better understand the difference between dbNSFP tools and LowMACA, we further annotated our dataset with four databases of manually curated variations in the human genome predicted as disease-associated. These four databases include two databases for disease-associated variations, Humsavar (UNIPROT database of human polymorphism at protein level, www.uniprot.org/docs/humsavar), clinvar version 20140929 [12] and two cancer-specific databases created by the Washington University, CiViC (Clinical Interpretation of Variants in Cancer, https://civic.genome.wustl.edu/#/home) and DoCM (Database of Curated Mutations http://docm.genome.wustl.edu/). CiViC and DoCM are not published yet. We then test the predictions of LowMACA against the union of Humsavar and clinvar and against the union of CiViC and DoCM. In both cases, there is a significant positive association (p-value << 0.01 and OR >>1) as reported in Table S6, meaning that predictions made by LowMACA are strongly in accordance with known results. Furthermore, we can appreciate a good overall recall and accuracy against these databases: 74% (32/43) and 95% (21/22) of recall for disease-

associated and cancer-associated variants respectively with an accuracy of 15% and 10%. We performed the same analysis against those variants evaluated as damaging by more than 50% of the dbNSFP tools. Although still positively associated to pathogenic variations, the results are less striking (p-value 3.6e-05 and 1.89e-03 for disease-associated and cancer-associated respectively). While still maintaining a good recall, the accuracy is extremely poor. This is not surprising since functional prediction tools are not intended to find mutations that actually occur in cancer or other diseases, but simply to assess if a possible variation could be harmful to the affected protein. LowMACA has the ability to discern those mutations that actually occur in patients, enhancing the accuracy of a true functional prediction.

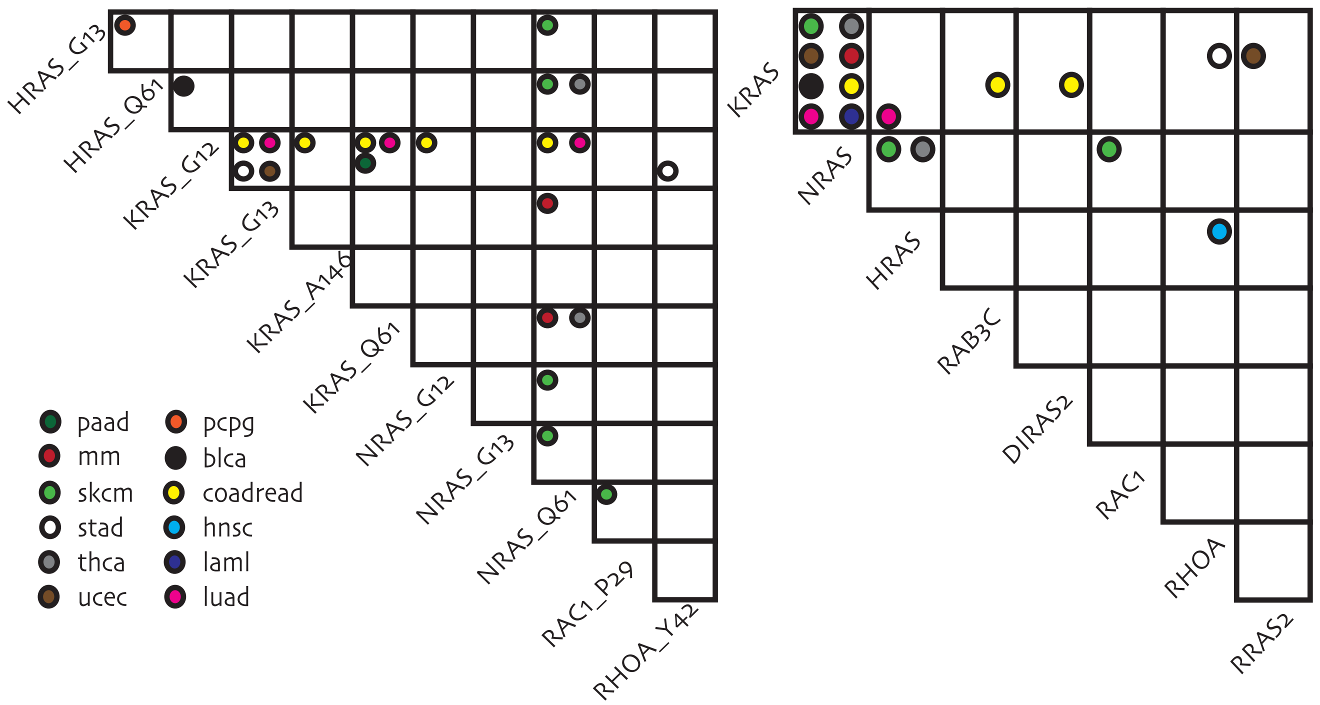

Figure S2
In this panel we show a plot representing the mutual exclusivity between mutations that fall in the same residue of individual proteins (left panel) and genes (right panel). Significant patterns are highlighted with the color corresponding to the tumor type where the exclusivity was found. We consider mutual exclusive the pairs with a corrected p-value below 0.05 using the R package *cooccur*. In the left panel we narrow down the patterns described in figure 1B, highlighting the major role of *KRAS* G12 and *NRAS* Q61. Notably, *RAC1* P29 and *RHOA* Y42 (described in the main text as potential new driver mutations) retain a pattern of mutual exclusivity with *NRAS* Q61 and *KRAS* G12, respectively. In the right panel it is possible to appreciate that mutual exclusivity between minor genes always occurs with the RAS trio. These genes cover many of the RAS subfamilies, in particular *RAB3C* (Rab subfamily), *DIRAS2* and *RRAS2* (Ras subfamily) and *RAC1* and *RHOA* (Rho subfamily).

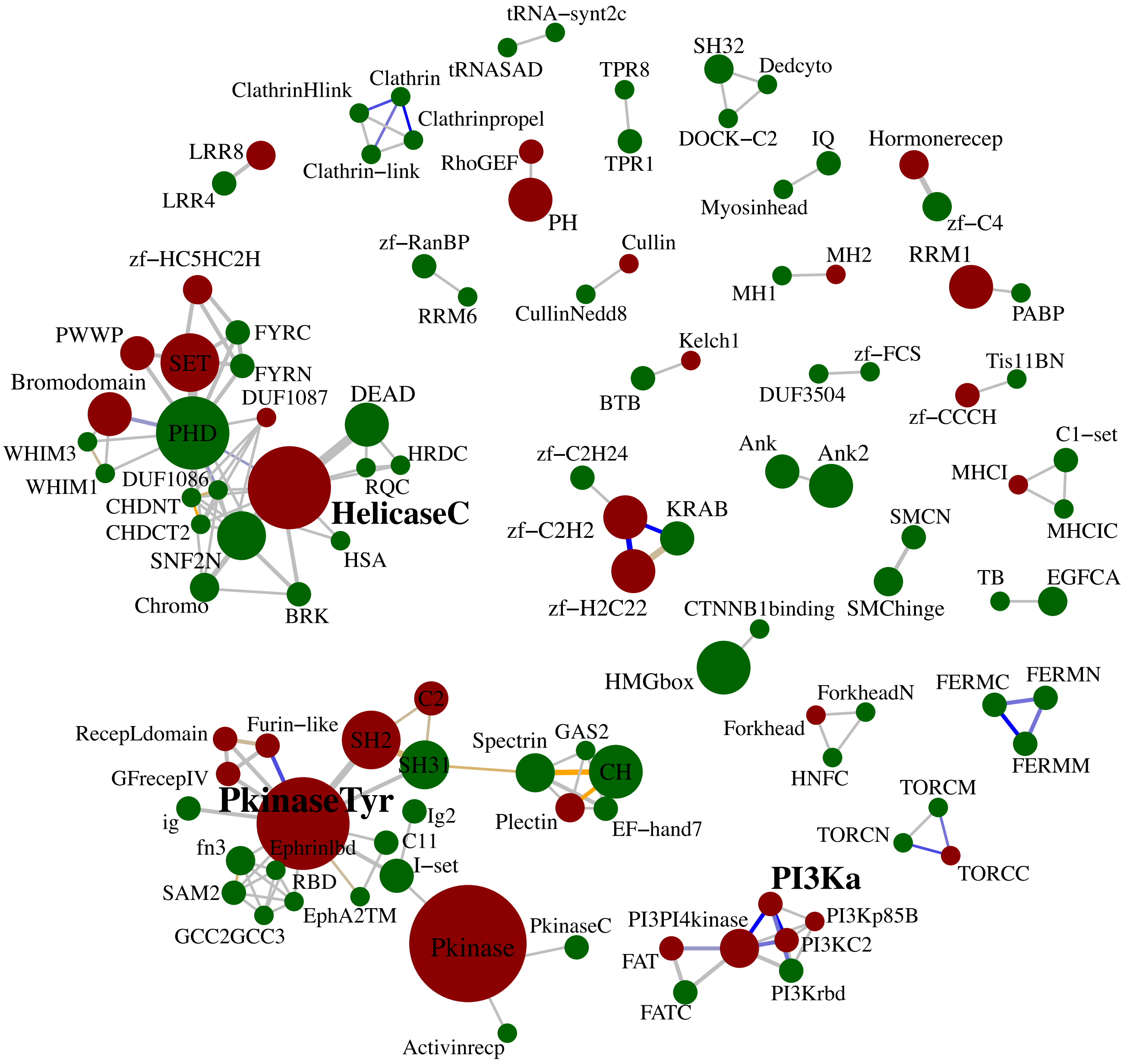

Figure S3

Complete Network of Pfam domains harbored by driver genes. 577 different domains are included in the list of 453 driver genes from Tamborero *et al.* that are represented by each circle in the plot. In red, we highlight those Pfams that harbor at least one significant hotspot, in green those that resulted not significant. An edge connects two domains if at least two genes harbor both the Pfam domains at the vertices. Blue edges are drawn if the domains are mutually exclusive (Fisher test < 0.05 is light blue, < 0.01 is deep blue and OR < 1), yellow if co-occurrening (Fisher test < 0.05 is light brown, < 0.01 is orange and OR > 1), grey if not significant. Excluding domains connected by only one gene, 110 out of the 577 are represented in this figure.

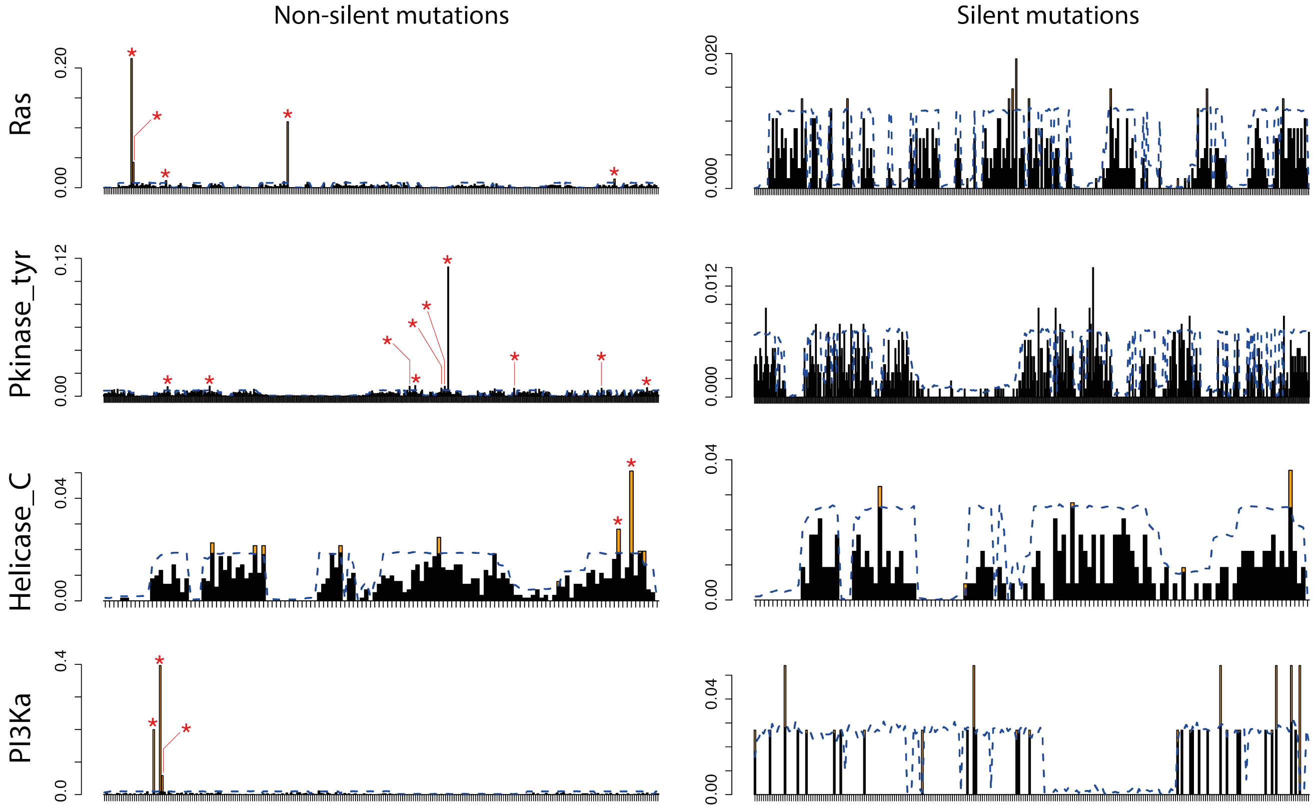

Figure S4

Barplots showing the stacking of silent and non-silent mutations within the 4 main Pfams discussed within the text (PF00071 - Ras superfamily, PF07714 - Pkinase_tyr, PF00271 - Helicase_C and PF00613 - PI3Ka). On the x-axis it is depicted the position in the global alignment, while on the y-axis the mutation frequency of ach position. The blue dashed line represent the threshold of significant mutation frequency, which is different for each position of the global alignment. Bars above the dashed blue line are significant in terms of their p-vlaue. Red asterisks highlight the residues that are significant after Benjamini-Hochberg procedure for multiple testing correction of p-value, which is performed only on conserved positions (see Methods). For silent-mutations analysis, a database was collected from TCGA original repositories and supplied as external repository for LowMACA analysis (see software Vignette for further information). The analysis shows that no hotspots are identified in any of the Pfams checked with the use of the silent mutations database, while at least two hotspots per Pfam are identified when the repository of non-silent mutations is used (canonical analysis). In particular, 5 hotspots are identified in Ras domain, 10 in Pkinase_tyr, 2 in Helicase_C and 3 in PI3Ka.

REFERENCES.

1. Liu X, Jian X, Boerwinkle E: **dbNSFP v2.0: A database of human non-synonymous SNVs and their functional predictions and annotations**. *Hum Mutat* 2013, **34**:2393–2402.

2. Gao J, Aksoy BA, Dogrusoz U, Dresdner G, Gross B, Sumer SO, Sun Y, Jacobsen A, Sinha R, Larsson E, Cerami E, Sander C, Schultz N: **Integrative Analysis of Complex Cancer Genomics and Clinical Profiles Using the cBioPortal**. *Sci Signal* 2013, **6**:pl1–pl1.

3. Cerami E, Gao J, Dogrusoz U, Gross BE, Sumer SO, Aksoy BA, Jacobsen A, Byrne CJ, Heuer ML, Larsson E, Antipin Y, Reva B, Goldberg AP, Sander C, Schultz N: **The cBio Cancer Genomics Portal: An open platform for exploring multidimensional cancer genomics data**. *Cancer Discov* 2012, **2**:401–404.

4. Wang K, Li M, Hakonarson H: **ANNOVAR: Functional annotation of genetic variants from high-throughput sequencing data**. *Nucleic Acids Res* 2010, **38**:1–7.

5. Adzhubei I a, Schmidt S, Peshkin L, Ramensky VE, Gerasimova A, Bork P, Kondrashov AS, Sunyaev SR: **A method and server for predicting damaging missense mutations**. *Nat Methods* 2010, **7**:248–249.

6. Reva B, Antipin Y, Sander C: **Predicting the functional impact of protein mutations: Application to cancer genomics**. *Nucleic Acids Res* 2011, **39**:1–14.

7. Schwarz JM, Cooper DN, Schuelke M, Seelow D: **MutationTaster2: mutation prediction for the deep-sequencing age.** *Nat Methods* 2014, **11**:361–2.

8. Kumar P, Henikoff S, Ng PC: **Predicting the effects of coding non-synonymous variants on protein function using the SIFT algorithm**. *Nat Protoc* 2009, **4**:1073–1081.

9. Dong C, Wei P, Jian X, Gibbs R, Boerwinkle E, Wang K, Liu X: **Comparison and integration of deleteriousness prediction methods for nonsynonymous SNVs in whole exome sequencing studies**. *Hum Mol Genet* 2015, **24**:2125–2137.

10. Chun S, Fay JC: **Identification of deleterious mutations within three human genomes**. *Genome Res* 2009, **19**:1553–1561.

11. Shihab H a., Gough J, Cooper DN, Stenson PD, Barker GL a, Edwards KJ, Day INM, Gaunt TR: **Predicting the Functional, Molecular, and Phenotypic Consequences of Amino Acid Substitutions using Hidden Markov Models**. *Hum Mutat* 2013, **34**:57–65.

12. Landrum MJ, Lee JM, Riley GR, Jang W, Rubinstein WS, Church DM, Maglott DR: **ClinVar: public archive of relationships among sequence variation and human phenotype**. *Nucleic Acids Res* 2014, **42**:D980–D985.
